# Cloning, Expression Profiling and Functional Analysis of *CnHMGS*, a Gene Encoding 3-hydroxy-3-Methylglutaryl Coenzyme A Synthase from *Chamaemelum nobile*

**DOI:** 10.3390/molecules21030316

**Published:** 2016-03-08

**Authors:** Shuiyuan Cheng, Xiaohui Wang, Feng Xu, Qiangwen Chen, Tingting Tao, Jing Lei, Weiwei Zhang, Yongling Liao, Jie Chang, Xingxiang Li

**Affiliations:** 1 School of Biology and Pharmaceutical Engineering, Wuhan Polytechnic University, Wuhan 430023, China; s_y_cheng@sina.com; 2 College of Horticulture and Gardening, Yangtze University, Jingzhou 434025, Hubei, China; wxhunique@foxmail.com (X.W.); chenqwx@foxmail.com (Q.C.); taotaoo2o3@foxmail.com (T.T.); 13872337364@163.com (J.L.); zww8312@163.com (W.Z.); liaoyongling@yeah.net (Y.L.); 3 Hubei Collaborative Innovation Center of Targeted Antitumor Drug, Jingchu University of Technology, Jingmen 448000, Hubei, China; 4 Medical School, Yangtze University, Jingzhou 434025, Hubei, China; lxx@yangtzeu.edu.cn

**Keywords:** *Chamaemelum nobile*, sesquiterpenoid biosynthesis, 3-hydroxy-3-methylglutaryl coenzyme A synthase, cloning, expression profile, functional complementation analysis

## Abstract

Roman chamomile (*Chamaemelum nobile* L.) is renowned for its production of essential oils, which major components are sesquiterpenoids. As the important enzyme in the sesquiterpenoid biosynthesis pathway, 3-hydroxy-3-methylglutaryl coenzyme A synthase (HMGS) catalyze the crucial step in the mevalonate pathway in plants. To isolate and identify the functional genes involved in the sesquiterpene biosynthesis of *C. nobile* L., a *HMGS* gene designated as *CnHMGS* (GenBank Accession No. KU529969) was cloned from *C. nobile*. The cDNA sequence of *CnHMGS* contained a 1377 bp open reading frame encoding a 458-amino-acid protein. The sequence of the CnHMGS protein was highly homologous to those of HMGS proteins from other plant species. Phylogenetic tree analysis revealed that CnHMGS clustered with the HMGS of Asteraceae in the dicotyledon clade. Further functional complementation of *CnHMGS* in the mutant yeast strain YSC6274 lacking HMGS activity demonstrated that the cloned *CnHMGS* cDNA encodes a functional HMGS. Transcript profile analysis indicated that *CnHMGS* was preferentially expressed in flowers and roots of *C. nobile*. The expression of *CnHMGS* could be upregulated by exogenous elicitors, including methyl jasmonate and salicylic acid, suggesting that *CnHMGS* was elicitor-responsive. The characterization and expression analysis of *CnHMGS* is helpful to understand the biosynthesis of sesquiterpenoid in *C. nobile* at the molecular level and also provides molecular wealth for the biotechnological improvement of this important medicinal plant.

## 1. Introduction

Roman chamomile (*Chamaemelum nobile* L.) is a perennial herb distributed in wild and cultivated habitats in Northern Africa, North America, Western Europe, and Asia. Customarily, Roman chamomile is regarded to be an antiseptic, vermifuge, antibiotic, bactericide, disinfectant and fungicide. This herb has been used for centuries as an anti-inflammatory medicine, antioxidant, mild sedative, mild astringent, antibacterial, antispasmodic agent, and healing medicine [1]. Oral dosage forms (infusions and decoctions) are used for the symptomatic treatment of gastrointestinal disorders and alleviation of functional digestive symptoms. External applications of roman chamomile extracts and lotions are often recommended as emollient or repellent in the treatment of skin disorders and for eye irritation or discomfort of various etiologies. In addition, the herb is used as an analgesic in diseases of the oral oropharynx, cavity, or both, as well as a mouthwash for oral hygiene [2].

Approximately 36 flavonoids and 28 terpenoids have been identified in chamomile [3]. The primary constitutes of the essential oils of Roman chamomile are sesquiterpene derivatives. The main components of the essential oil of Roman chamomile were found to β-pinene, germacrene D, α-bisabolol, and chamazulene [4,5], which are sesquiterpenoid and effective anti-inflammatory and anti-allergic substances [6]. Although extensive studies have carried out regarding the pharmacological importance of the essential oil constituents, little is known about the genes responsible for biosynthesis of these sequiterpenoids.

**Scheme 1:**
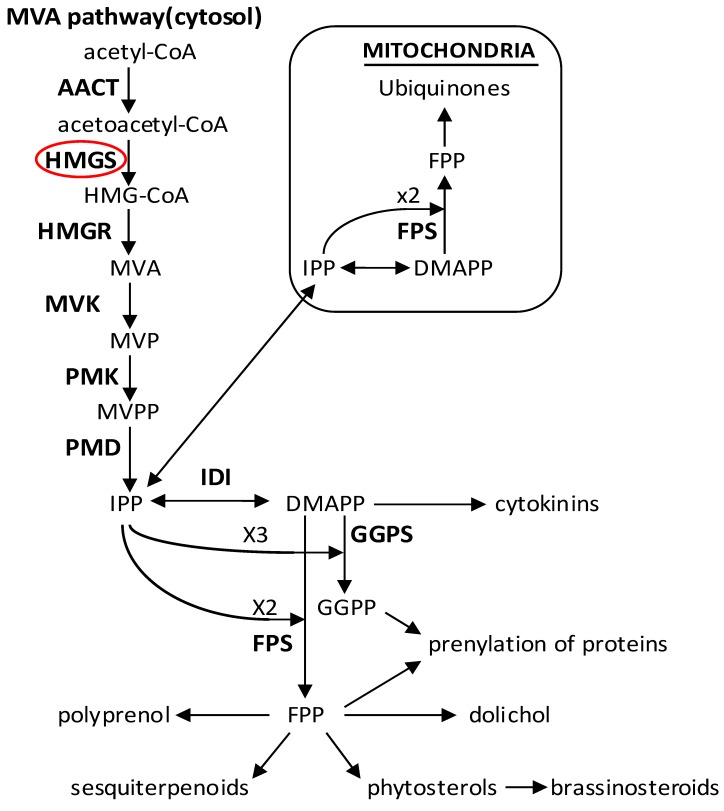
Isoprenoid biosynthetic MVA pathway in the plant cell.

Terpenoids are synthesized in plants through two pathways, namely, the mevalonate (MVA) and 2*C*-methyl-d-erythritol 4-phosphate ones [7]. As an important condensing enzyme in the MVA pathway, 3-hydroxy-3-methylglutaryl coenzyme A synthase (HMGS) catalyzes the condensation of acetyl-CoA and acetoacetyl-CoA to generate HMG-CoA, which is further converted into MVA by 3-hydroxy-3-methylglutaryl coenzyme A reductase (HMGR) (Scheme 1). The isopentenyl pyrophosphate (IPP) of the C5 skeleton is generated by the pyrophosphorylation and decarboxylation of MVA; common precursors are supplied for the synthesis of terpenoid compounds such as mono-, sesqui-, di-, and triterpenoids. Normally, sesquiterpenoids are synthesized from MVA pathway in the cytosol [8]. Given its importance in terpenoid biosynthesis, some *HMGS* genes have been studied for its function in the MVA pathway. Since the first plant *HMGS* gene was cloned from *Arabidopsis thaliana* in 1995 [9], numerous papers have reported cloning and characterization of *HMGS* genes from almost 40 plants [10,11]. Although *C. nobile* is an important medicinal plant, few studies focused on identifying the enzymes or genes involved in sesquiterpenoid biosynthesis. To unveil the overall biosynthetic pathway of terpenoids in *C. nobile*, each gene involved in this pathway should be isolated and characterized. In this paper, we report the cloning of the full-length cDNA sequence of the *HMGS* gene from *C. nobile* for the first time. We also present the characterization, evolution, transcription profiling and functional analyses of *CnHMGS*.

## 2. Results and Discussion

### 2.1. Cloning and Characterization of CnHMGS

**Figure 1:**
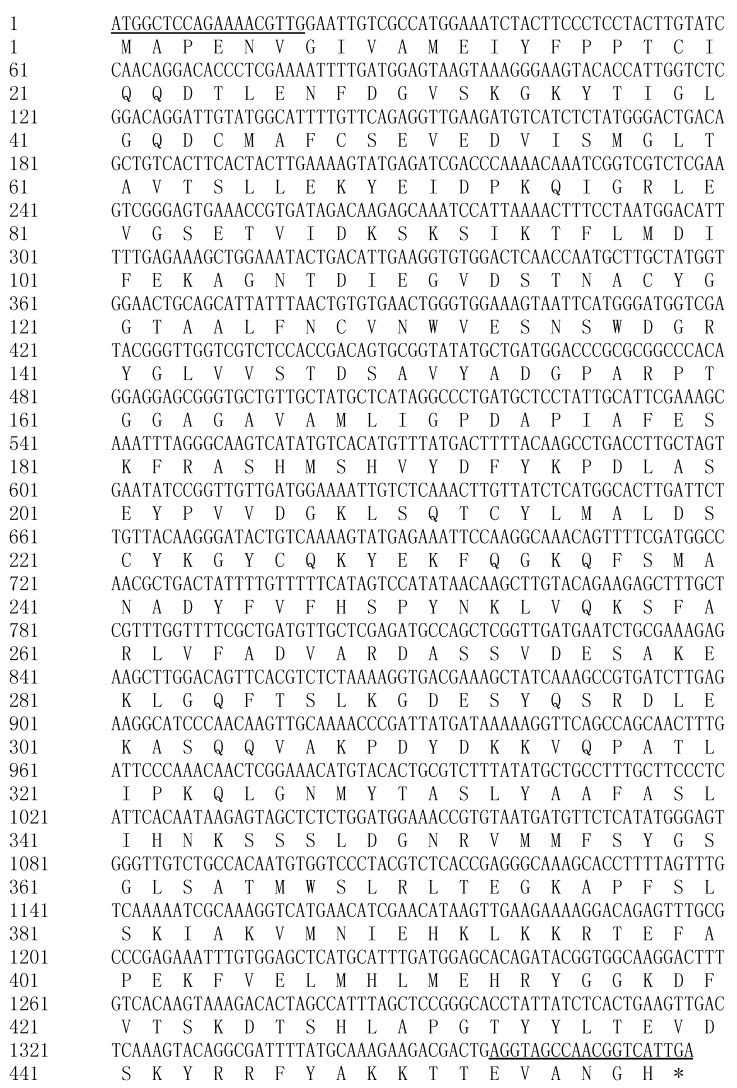
The nucleotide acid sequence and deduced amino acid sequence of *CnHMGS*. The underline for specific primers CnHMGSR and CnHMGSF.

The PCR products were sequenced, and results showed that the cDNA sequence of the PCR products was 1377 bp. The results of BLASTN analysis on NCBI showed that the cDNA sequence of *CnHMGS* had a high similarity to those of other *HMGS* genes. The nucleotide sequence of *CnHMGS* was 94.0%, 81.0%, 79.0%, 79.0%, and 78.0% identical to those of the *HMGS* genes from *Artemisia annua*, *Platycodon grandiflorus*, *Panax ginseng*, *Camptotheca acuminata*, and *Vitis vinifera*, respectively (Table 1). The result indicates that the gene we cloned is a member of the *HMGS* gene family. Therefore, this gene was designated as *CnHMGS* (GenBank Accession No. KU529969). As shown in Figure 1, the nucleotide sequence of the *CnHMGS* gene contained a 1374 bp ORF that encodes a predicted protein sequence of 458 amino acid residues.

### 2.2. Characterization of the Deduced CnHMGS Protein

The deduced CnHMGS protein contained 458 amino acids. Computer pI/Mw Tool was used to calculate the molecular weight and isoelectric point (pI) of the deduced CnHMGS protein, which were predicted to be 50.1 kDa and 6.05, respectively. The multiple alignments showed that the deduced CnHMGS had a high homology with the HMGSs from other plants.

**Figure 2:**
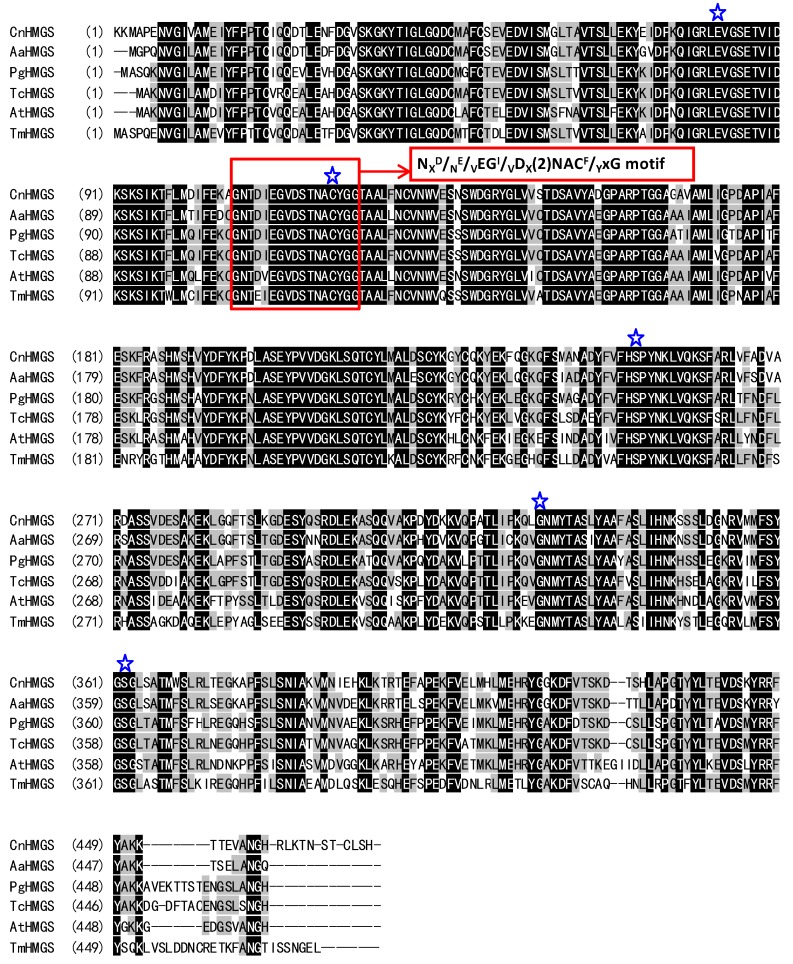
The multiple alignments of CnHMGS amino acid sequence with other HMGS proteins. The completely identical amino acids are indicated with white foreground and black background. The conserved amino acids are indicated with black foreground and grey background. Non-similar amino acids are indicated with black foreground and white background. The conservative motif, “NxD/NE/VEGI/VDx(2)NACF/YxG” responsive for catalytic is boxed. The active sites is indicated with star. The species, protein names and GenBank accession numbers are as following: *C. nobile*: CnHMGS; *Artemisia annua*: AaHMGS (ACY74339); *Panax ginseng*: PgHMGS (ADI80347); *Theobroma cacao*: TcHMGS (XP_007040101); *Arabidopsis thaliana*: AtHMGS (CAA58763.1); *Taxus media*: TmHMGS (AAT37206.1).

The CnHMGS protein showed 81.5%, 80.2%, 80.6%, 80.7%, 80.4%, and 89.7% identities to the counterparts of *Salvia miltiorrhiz*a, *Ricinus communis, Theobroma cacao, Nicotiana langsdorffii* × *Nicotiana sanderae*, *P. ginseng*, and *A. annua*, respectively. As shown in Figure 2, the polypeptide chain of CnHMGS contained three domains: the *N*-terminus, catalytic region, and the *C*-terminus. The CnHMGS protein contained the conserved motif “NxD/NE/VEGI/VDx(2)NACF/YxG” [12] and five conserved active sites of amino acids, namely, Glu82, Cys120, Ser251, Gly328, and Ser362 [13].

### 2.3. Three-Dimensional Model Analysis

**Figure 3:**
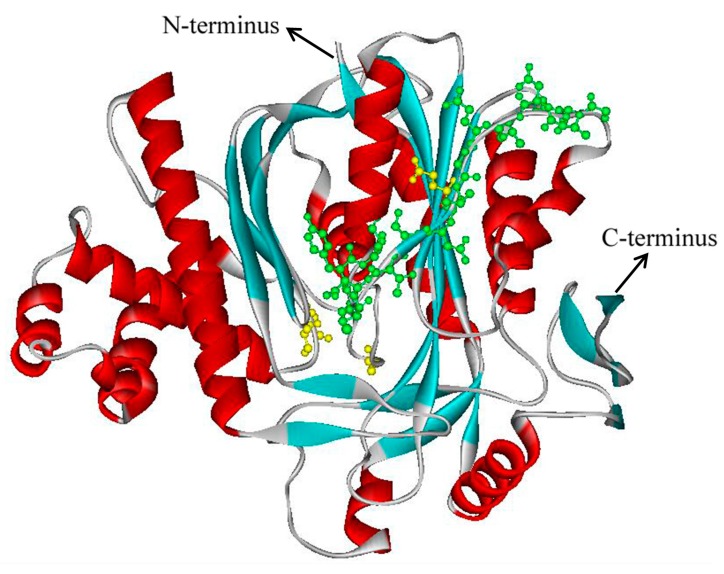
The three-dimensional model of CnHMGS. The α-helices are indicated by helices in red and β-sheets are indicated by patches in blue. Turns and loops are indicated by lines. Conserved motif and active sites are indicated by ball and stick in green and yellow, respectively.

In order to better understand the CnHMGS protein, a comparative modeling of the 3D structure of CnHMGS was generated based on the highest query coverage of the template *Brassica juncea* HMGS (2f82.1.A) with SWISS-MODEL [14]. The 3D structural analyses were performed with Weblab Viewerlite. As shown in Figure 3, the predicted 3D model revealed CnHMGS consist of two structural regions referred as the lower and upper regions, similar to the HMGSs from other plants [9]. The conserved motifs GNTDIEGVDSTNACYGG and “NxD/NE/VEGI/VDx(2)NACF/YxG” formed the core of the enzyme structure. All the conserved motifs and active sites were localized in a five-layered core structure of upper region.

### 2.4. Molecular Evolution Analysis

**Figure 4:**
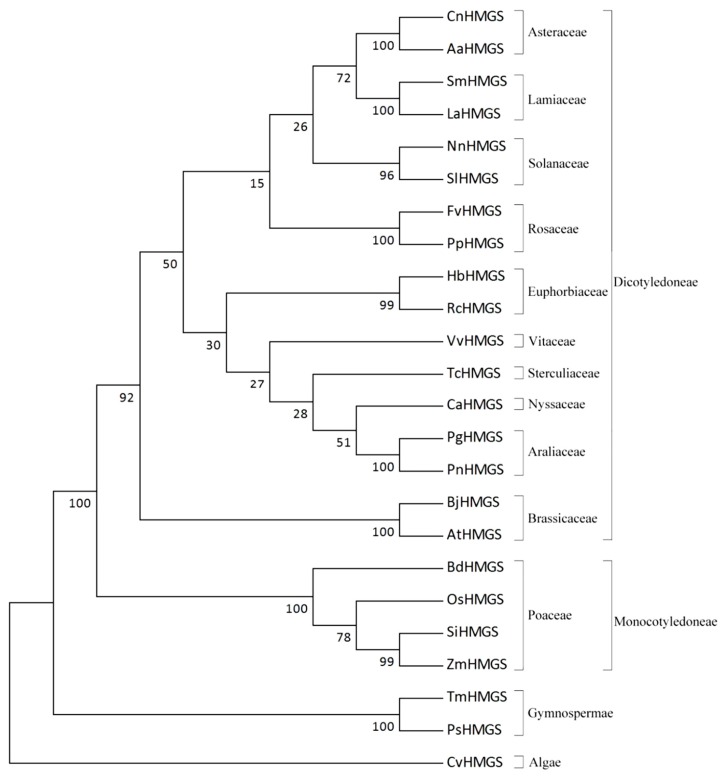
Phylogenetic tree analysis of protein encoded by *HMGS* genes. The species, protein names and GenBank accession number are as following: *C. nobile*: CnHMGS; *Camptotheca acuminata*: CaHMGS (ACD87446.1); *Panax ginseng*: PgHMGS (ADI80347); *Vitis vinifera*: VvHMGS (CBI34763.3); *Theobroma cacao*: TcHMGS (XP_007040101); *Hevea brasiliensis*: HbHMGS (AAK73854.1); *Ricinus communis*: RcHMGS (EEF51079.1); *Fragaria vesca* subsp. vesca: FvHMGS (XP_004298742.1); *Nicotiana langsdorffii* x *Nicotiana sanderae*: NnHMGS (ABV02025.1); *Salvia miltiorrhiza*: SmHMGS (ACV65039.1); *Artemisia annua*: AaHMGS (ACY74339); *Brassica juncea*: BjHMGS (AAF69804.1); *Arabidopsis thaliana*: AtHMGS (CAA58763.1); *Brachypodium distachyon*: BdHMGS (XP_003574875.1); *Oryza sativa*: OsHMGS (EAZ09792.1); *Setaria italica*: SiHMGS (XP_004957395.1); *Zea mays*: ZmHMGS (DAA40580.1); *Taxus media*: TmHMGS (AAT37206.1); *Pinus sylvestris* L: PsHMGS (CAA65250.1); *Chara vulgaris*: CvHMGS (ABO27206.1); *Solanum lycopersicum*: SlHMGS (NP_001234846); *Lavandula angustifolia*: LaHMGS (AGQ04158); *Panax notoginseng*: PnHMGS (AIK21781); *Prunus persica*: PpHMGS (EMJ11192). The bars represent evolutionary distance. The reliability of the tree is measured by bootstrap analysis with 100 replicates.

In order to study the evolutionary relationships in CnHMGS and HMGS proteins from other plants, we selected the typical HMGS proteins from GenBank. We constructed a phylogenetic tree using the software MEGA 5.0 with NJ method to analyze the molecular evolution of CnHMGS on the basis of the four groups (Dicotyledoneae, Monocotyledoneae, Gymnospermae, and Algae) and eleven families of angiosperms. As shown in Figure 4, CnHMGS belonged to Dicotyledoneae in the branch of Dicotyledoneae, Monocotyledoneae, Gymnospermae, and Algae; the CnHMGS protein had the closest relationship to AaHMGS of *A. annuain* Asteraceae. These results suggest that CnHMGS shares a common evolutionary with other plant HMGS proteins based on conserved structure and sequence characteristics, such as amino acid homologies and conserved motifs, respectively.

### 2.5 Functional Complementation of CnHMGS in Saccharomyces cerevisiaes

**Figure 5:**
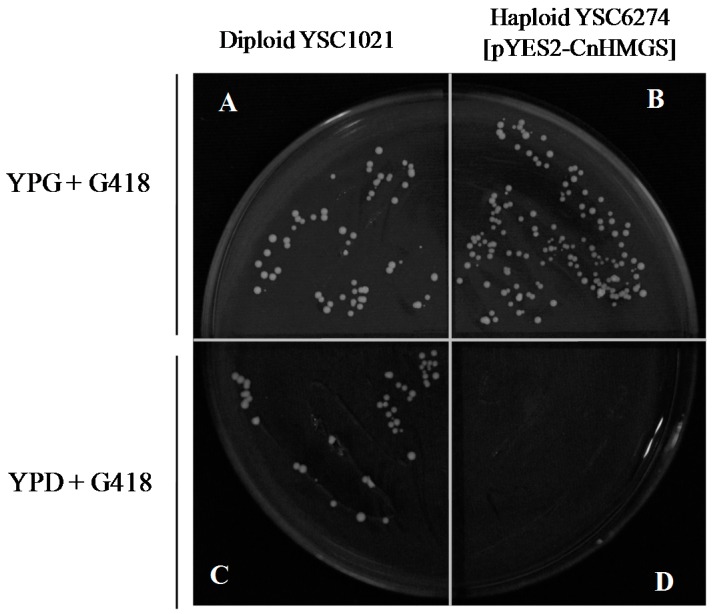
Functional complementation for the growth of the yeast strain YSC6274 *by CnHMGS*. (**A**) Diploid YSC1021 strain on YPG + G418 medium grew within 2 days; (**B**) Haploid YSC6274 strain containing *pYES2-CnHMGS* on YPG + G418 medium grew within 2 days; (**C**) Diploid YSC1021 strain on YPD + G418 medium grew within 2 days; (**D**) Haploid YSC6274 strain containing *pYES2-CnHMGS* on YPD + G418 medium failed to grow.

**Figure 6:**
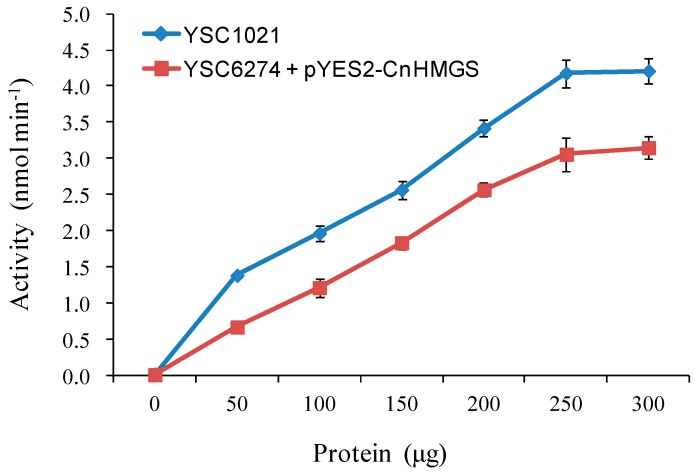
The activity of HMGS in crude extract at various protein concentrations. The YSC1021 and YSC6274 + pYES2-CnHMGS are enzyme activity from crude extract of yeast strain YSC1021 and YSC6274 harboring pYES2-CnHMGS, encoding native yeast HMGS and recombinant CnHMGS, respectively.

To access the function of *CnHMGS*, its cDNA was expressed in the mutant *Saccharomyces cerevisiaes* strain YSC6274 which lacks HMGS1 and HMGS2 activity and requires MVA for growth [15]. We successfully constructed the expressing vector pYES2-CnHMGS and the *CnHMGS* gene was driven by a galactose-dependent promoter. Wild type *S. cerevisiaes* strain YSC1021 were able to grow on either YPG expression medium (Figure 5A) or YPD non-expression medium (Figure 5C). Only the transformed yeast YSC6274 strain with pYES2-CnHMGS could grew on YPG expression medium (Figure 5B), but not on YPD non-expression medium (Figure 5D). The expression of *CnHMGS* can rescue the functional defect of the HMGS knockout yeast. Our data indicates that CnHMGS exhibits HMGS activity. In addition, the expressed recombinant CnHMGS from transformed strain YSC6274 at various protein concentration showed significantly lower activity than HMGS from strain YSC1021 in the range of protein in the assay condition (Figure 6), suggesting that CnHMGS protein is less active than native yeast HMGS enzyme.

### 2.6. Transcript Level of the CnHMGS in C. nobile

**Figure 7:**
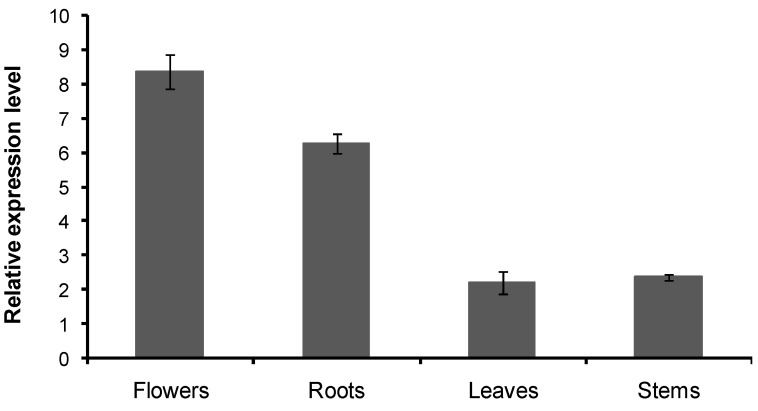
Spatial expression analysis of *CnHMGS* in stem, root, leaf and flower by Real Time-PCR. Data are means ± SD from triplicate experiments (*n* = 3).

As shown in Figure 7, the *CnHMGS* gene was expressed constitutively in all tissues examined with different levels. The highest transcript level of *CnHMGS* was observed in flowers, followed by roots, whereas a significantly lower transcript accumulation was detected in the steam and leaf. The results reveal that *CnHMGS* has a preferential expression pattern in some organs, such as flower and root.

### 2.7. Expression Profile of CnHMGS under Induction of MeJA and SA Elicitor

**Figure 8:**
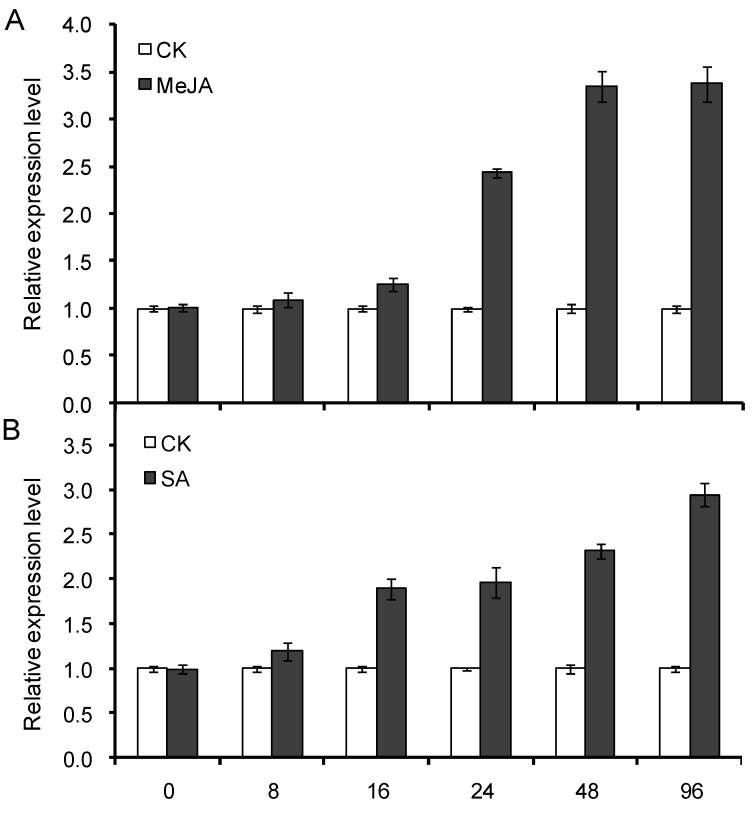
The *CnHMGS* expression level changes in *C*. *nobile* by MeJA (**A**) and SA (**B**). For each treatment, the expression levels are normalized to *Cn18S* gene. The gene expression level of Control (CK) was set to 1, and those of treatments were accordingly accounted and presented as the relative fold changes. Data are means ± SD from triplicate experiments (*n* = 3).

To understand the expression pattern of *CnHMGS*, *C*. *nobile* seedlings were treated with the signal molecules MeJA and SA and measured mRNA levels by qRT-PCR analysis. The expression of *CnHMGS* was strongly induced by SA, reaching the highest level (3.37-fold as compared with control) at 96 h after treatment (Figure 8A). Figure 8B showed that the *CnHMGS* expression was effectively induced by SA. The *CnHMGS* transcript level reached the highest level (2.95-fold as compared with control) at 96 h post-treatment. The results suggest that the expression of *CnHMGS* might be involved in the processes regulated by MeJA and SA in *C*. *nobile*.

### 2.8. Discussion

In recent years, there has been a remarkable progress in the understanding of the molecular regulation of sesquiterpenoid biosynthesis in plant [16,17]. As an important enzyme in the sesquiterpenoid biosynthetic pathway, HMGS has been shown to be an rate-limiting enzyme which catalyze the important step in the MVA biosynthesis pathway [10]. Since Rudney and Ferguson [14] first showed that HMGS participates in the synthesis of polyisoprene, other scientists confirmed that HMGS is involved in the second step of catalysis via the MVA pathway [18,19,20]. The rubber produced by *Hevea brasiliensis* is an important industrial material and a type of terpenoid. Suwanmanee *et al.* [21] cloned a *HMGS* gene from *H. brasiliensis* at the first time. Further study indicated that the mRNA levels and activity of HMGS in *H. brasiliensis* are closely related to the accumulation of rubber in the plants.

Recently, Ren *et al.* [12] also demonstrated *HMGS* is a key gene involved in the biosynthesis of triterpene ganoderic acid in *Ganoderma lucidum* by overexpression analysis of the *GlHMGS* gene. Given its importance in terpenoid biosynthesis, *HMGS* is studied for its function in the MVA pathway. However, few literature studies have described the enzymes or genes including *HMGS* in the sesquiterpenoid biosynthetic pathway in *C. nobile*. In the present study, we attempted to dissect the molecular biology of the sesquiterpenoid biosynthetic pathways in *C. nobile* by cloning and characterizing the full-length cDNA of *CnHMGS*.

In this work, a 1374 bp full-length cDNA of the gene *CnHMGS* was isolated from *C. nobile*. The deduced CnHMGS protein contained 458 amino acids and weighed 50.1 kDa, which is consistent with the fact that the plant HMGS protein is generally composed of 460–500 amino acid residues and has a relative molecular mass of 50–60 kDa [22,23,24,25,26]. The multiple alignments showed that the deduced CnHMGS protein sequence had high similarity to other plant HMGSs. Molecular evolution analysis revealed that the CnHMGS protein had the closest relationship to AaHMGS of *A. annua*, which is similar to the HMGS sequences of Phycophyta and higher plants that belong to two separate branches. This trend indicates that *HMGS* is conserved in terms of evolutionary origin across different plants and shows the conservation of amino acid sequences and functional domains. Moreover, protein motif analysis showed that the CnHMGS contained the conserved motif “NxD/NE/VEGI/VDx(2)NACF/Yx G” and had five active sites: Glu82, Cys120, Ser251, Gly328, and Ser36. Our results are similar to those observed in most HMGS proteins with the conserved motif “NxD/NE/VEGI/VDx(2)NACF/YxG”, which is considered important for HMGS function. This motif is localized at the entrance of the active site and plays an important role in controlling the catalysis of substrates by HMGS. Mutation of this motif decreases the catalytic activity of the enzyme or leads to the formation of abnormal products. In addition, three conserved amino acid residues presented in CnHMGS, namely, Cys129, His264, and Asn326, are essential for the catalytic activity of the enzyme [27]. Therefore, CnHMGS is likely to have a similar catalytic activity in the MVA pathway in *C. nobile*. CnHMGS complement assays revealed that the expression of CnHMGS provides the basic material for yeast survival, thereby confirming the catalytic function of CnHMGS. Furthermore, our results indicated CnHMGS possessed apparent activity but significantly lower than that of native yeast HMGS.

*C. nobile* is rich in active ingredients and has remained one of the most popular herbs since ancient times. Usually the flowers of chamomile plants are used as the source for medicinal purposes. A recent chemical analysis of chamomile has shown that chamazulene is the most abundant terpenoids in the flower (28%–31% of total terpenoids) [4]. Besides flowers, chamomile roots are also rich in essential oil containing sesquiterpenes, hydrocarbons and alcohols [28]. Therefore, it is interesting to determine whether it is therefore predicted that the spatial expression profile of *CnHMGS* is positively correlated with the active principle content in different tissues of *C. nobile*. Several studies showed that the expression pattern of *HMGS* in plant tissues greatly varies across different plants. For example, *HMGS* is mainly expressed in the needles, stems, hypocotyls, and cotyledons but is seldom expressed in the roots of *C. acuminata* [26] and *Taxus media* [29]. By contrast, *HMGS* is constitutively expressed in the leaf, stem, and root of *S. miltiorrhiz*a [25]. Alex *et al.* [30] studied the developmental expression pattern of *HMGS* in the flower, seed, and seedling of *B. juncea* and found that *HMGS* expression is the highest at earlier stages in these plant parts. Our results revealed that the expression level of *CnHMGS* is significantly higher in the flower and root than in other tissues. This trend is consistent with the chemical constituent analysis from earlier reports [4,28,31,32,33], which showed that significant higher contents of the major sesquiterpenoids were observed in the flowers and roots than other part of chamomile. These results support a correlation between the transcript level of *CnHMGS* and the content of sesquiterpenoids, suggesting that *CnHMGS* plays an important role in the production of sesquiterpenoids in *C. nobile*.

MeJA and SA are phytohormones and signal molecules that regulate the response of plants against both abiotic and biotic stresses, such as UV radiation, ozone exposure, herbivore and pathogen attacks [34,35,36]. In addition, MeJA and SA were proved as the elicitors triggering the pathway of secondary metabolism in plant [37,38]. It has been demonstrated that the expression of *HMGS* genes was induced by MeJA and SA in *G. lucidum* [12], *S. miltiorrhiza* [25], *C. acuminata* [26], and *Tripterygium wilfordii* [39]. The upregulation of a gene under the influence of MeJA and SA indicates the involvement of this gene in such response. Our qRT-PCR experiments also clearly showed an increase in the transcript level of *CnHMGS* from *C. nobile* seedlings under MeJA and SA treatments. Taken into account HMGS as one of key enzymes involved in sesquiterpene biosynthesis in plant, the expression profile of *CnHMGS* suggests that MeJA and SA treatments might be an effective approach to induce the higher production of sesquiterpene in *C. nobile*. However, the roles and regulatory mechanisms of *HMGS* genes in the biosynthesis of active constituents, especially the sesquiterpene with a high anti-inflammatory activity, needs to be further investigated in *C. nobile*.

## 3. Experimental Section

### 3.1. Plant Materials

Roman chamomile (*C. nobile*) was grown in a phytochamber maintained with a 16 h light/8 h dark photoperiod at 23 °C. The roots, stems, leaf, and flower samples were harvested from 8-week-old *C. nobile*. To test the spatial expression of *CnHMGS*, the leaves, stems, roots, and flowers of *C. nobile* seedlings were collected. All the samples were quickly frozen in liquid nitrogen and preserved at −80 °C until further analyses. 8-week-old seedlings of *C. nobile* were sprayed with the solution of 100 μM methy jasmonate (MeJA) or salicylic acid (SA), respectively, with water as control for each treatment. MeJA was first dissolved in a small of ethanol, and then diluted with distilled water to form final concentration 100 μM. SA was dissolved in distilled water to form the SA solution. 1.0 g leaves of seedlings treated with SA and MeJA was harvested, respectively, at 0, 8, 16, 24, 48, 96 h.

### 3.2. Cloning of the Full-Length cDNA of CnHMGS

Total RNA was isolated from *C. nobile* using the CTAB method [40]. The concentration and quality of the RNA were measured via spectrophotometry and agarose gel electrophoresis. The primers CnHMGSR (5′-ATGGCTCCAGAAAACGTTG-3′) and CnHMGSF (5′-TCAATGACCGTTGGCTACCT-3′) were designed and synthesized (Shanghai Sangon, Shanghai, China) according to the gene annotation of *C. nobile* in the transcriptome database.

One-step RT-PCR was performed, and a 1377 bp fragment was amplified with the one-step RT-PCR kit (Dalian TaKaRa, Dalian, China) under the following conditions: 94 °C for 3 min; 33 cycles of amplification at 94 °C for 20 s, 56 °C for 40 s, and 72 °C for 60 s; and a final extension at 72 °C for 7 min. The PCR products were purified, ligated into the pMD19-T vector (Dalian TaKaRa), and then cloned into the *Escherichia coli* strain DH5a before sequencing. Subsequent BLAST results confirmed that the obtained product was the nucleotide sequence of the *CnHMGS* gene.

### 3.3. Bioinformatics Analysis and Molecular Evolution Analyses

The obtained nucleotide sequence and deduced amino acid sequence were compared by a BLAST database search (http://www.ncbi.nlm.nih.gov). The molecular weight and isoelectric point (pI) of the deduced CnHMGS protein were computed with the Compute pI/Mw tool (http://web.expasy.org). Multiple sequence alignment was performed with the Vector NTI suite 10.0 program (Invitrogen, Paisley, UK). A phylogenetic tree was constructed with CLUSTALX 2.0 (Conway Institute UCD Dublin, Dublin, Ireland, http://www.clustal.org) and MEGA 4.0 (Biodesign Institute, Tempe, AZ, USA, http://www.megasoftware.net). The reliability of the tree was measured by bootstrap analysis with 100 replicates.

### 3.4. CnHMGS Transcript Analysis by Real-Time PCR

The expression level of *CnHMGS* was determined by real-time PCR (qRT-PCR). A 1 μg aliquot of the total RNA was used as the template for qRT-PCR. qRT-PCR was performed using a Bio-Rad Mini OpticonTM Real-time PCR Mini Cycler (BioRad, Hercules, CA, USA) with SYBR Premix Ex Taq™ II Kit (Dalian TaKaRa) according to the method of Xu *et al.* [41]. The primers for *CnHMGS* (CnHMGSR1: 5′-GGTCTCGGACAGGATTGTATGG-3′, CnHMGSF1: 5′-TGTAACAAGAATCAAGTGCC-3′), and housekeeping gene *18S* gene (18SF:5′-ACGAGACCTCAGCCTGCTAACT-3′, 18SR: 5′-CCAGAACATCTAAGGGCATCACA-3′) were designed according to the Sequence Detection System software. Raw data were analyzed with MiniOpticon™ Real-time PCR Detection system, and expression level was normalized into *18S* gene to minimize the variation in the cDNA template levels. qRT-PCR data were technically replicated with error bars, representing mean ± SD (*n* = 3).

### 3.5. Functional Complementation of CnHMGS in Yeast

The coding region of the *CnHMGS* ORF was amplified via PCR with the following primers: HMGS_CS (5′-CCCAAGCTTATGGCTCCAGAAAACGTTG-3′), which contained the *Hind*III restriction site, and HMGS_CA (5′-CGGAATTCTCAATGACCGTTGGCTACCT -3′), which contained the *Eco*RI restriction site. The product and pYES2 vector (Invitriogen, Carlsbad, CA, USA) were digested with *Hind*III and *Eco*RI, respectively, and then ligated. Positive clones were confirmed by PCR and sequencing. The constructed pYES2-CnHMGS plasmids were extracted for transforming.

The wild-type *S. cerevisiae*s train YSC1021 is a diploid yeast. The haploid *S. cerevisiae*s strain YSC6274 lacking the *HMGS* allele was purchased from the Open Biosystems Yeast Knock Out Strain Collection (Open Biosystems, Huntsville, AL, USA). The pYES2-CnHMGS plasmid was transformed into YSC6274 with the Frozen-EZ Yeast Transformation II Kit (ZymoResearch, Orange, CA, USA). The transformants were spotted on SC (-Ura) medium (6.7% yeast nitrogen base without amino acid, 2% galactose). Positive clones were further confirmed through PCR. Subsequently, the transformed diploid cells were induced to sporulate and form haploid cells containing pYES2-CnHMGS. The diploid *S. cerevisiae* strain YSC1021 and haploid strain YSC6274 cells were separately grown on YPD + G418 and YPG + G418 media to compare their growth conditions.

### 3.6. HMGS Enzyme assay

The cultures of *S. cerevisiae* strain YSC1021 and strain YSC6274 harboring the pYES2-CnHMGS plasmids were incubated in YPG + G418 liquid media for 48 h. The crude protein was extracted from yeast cultures using a modified alkaline extraction method [42]. HMGS activity was determined by the method described by Sirinupong *et al*. [13].

## 4. Conclusions

We have successfully cloned and characterized for the first time the gene *CnHMGS* encoding HMGS, which is involved in sesquiterpene biosynthesis in the medicinal plant *C. nobile*. Multiple sequence alignment of the deduced CnHMGS protein showed the highest identity to that of *A. annua.* CnHMGS was shown to complement yeast mutants defective in HMGS. *CnHMGS* was preferentially transcribed in flowers and roots. The expression of *CnHMGS* was upregulated by signal molecules (MeJA and SA), which indicated that it was inducible and might be involved in signal molecule response to environmental stimuli. The present study is helpful to understand the sesquiterpene in *C. nobile* at the molecular level. Further studies on the identification of the genes related to the terpenoid biosynthesis of this plant would be useful for understanding sesquiterpene biosynthesis and would provide molecular resources for the biotechnological improvement of this important medicinal plant.

## Figures and Tables

**Table 1:** Nucleotide sequence of *CnHMGS* similarity to the *HMGS* genes from other plant species.

Species	Accession No.	Homology
*Artemisia annua*	GQ468551	94.0%
*Platycodon grandiflorus*	KC439366	81.0%
*Panax ginseng*	GU565098	79.0%
*Camptotheca acuminata*	EU677841	79.0%
*Vitis vinifera*	FQ383761.1	78.0%
